# Inertia Coupling Analysis of a Self-Decoupled Wheel Force Transducer under Multi-Axis Acceleration Fields

**DOI:** 10.1371/journal.pone.0118249

**Published:** 2015-02-27

**Authors:** Lihang Feng, Guoyu Lin, Weigong Zhang, Dong Dai

**Affiliations:** School of Instrument Science and Engineering, Southeast University, Nanjing, China; Coastal Carolina University, UNITED STATES

## Abstract

Wheel force transducer (WFT), which measures the three-axis forces and three-axis torques applied to the wheel, is an important instrument in the vehicle testing field and has been extremely promoted by researchers with great interests. The transducer, however, is typically mounted on the wheel of a moving vehicle, especially on a high speed car, when abruptly accelerating or braking, the mass/inertia of the transducer/wheel itself will have an extra effect on the sensor response so that the inertia/mass loads will also be detected and coupled into the signal outputs. The effect which is considered to be inertia coupling problem will decrease the sensor accuracy. In this paper, the inertia coupling of a universal WFT under multi-axis accelerations is investigated. According to the self-decoupling approach of the WFT, inertia load distribution is solved based on the principle of equivalent mass and rotary inertia, thus then inertia impact can be identified with the theoretical derivation. The verification is achieved by FEM simulation and experimental tests. Results show that strains in simulation agree well with the theoretical derivation. The relationship between the applied acceleration and inertia load for both wheel force and moment is the approximate linear, respectively. All the relative errors are less than 5% which are within acceptable and the inertia loads have the maximum impact on the signal output about 1.5% in the measurement range.

## Introduction

When the vehicle is moving on the road, three-axis forces and three-axis torques are applied to the wheel, which is longitudinal force *F*_*x*_, lateral force *F*_*y*_, vertical force *F*_*z*_, heeling moment *M*_*x*_, twist torque *M*_*y*_, and aligning torque *M*_*z*_, respectively. The interaction between the tyre and ground is represented by these forces, and therefore, sensing the wheel forces/torque is quite significant in vehicle testing field [1–5]. To gain the time histories of these forces, the famous multi-axis wheel force transducer (WFT) [6–12] which offers the capability of acquiring load data at the spindle of a vehicle, has been promoted extremely by researchers and engineers with great interests.

Generally, as an on-board instrument for wheel forces measurement dynamically, the WFT consists of a multi-axis force sensor (MFS) [13–20], in which an elastic body can deform under the applied forces. Measurement of the elastic deformations by appropriate transducers yields electrical signals from which the force components are derived. From all beginnings on the multi-axis force sensors, researchers are making great efforts to reduce the cross coupling errors in dimensions. Techniques are mainly covered by two categories, one is the structure optimization [15–18] that force/moment self-decoupled transducer is designed to eliminate the coupling interferences, the other is the signal decoupling [11–12, 19–20] that multi-axis interferences are reduced by using various data algorithms and methods. Apparently, a well-designed structure with optimization is the primary target while the signal processing is always acting on the basis of structure decoupling. In general use, these multi-axis sensors are small and the structure analysis is under approximate static state, which means the inertia/mass of the sensor itself exerts little influence on the sensor response or the signal processing is fully able to cope with it. Reports have shown that a great number of these multi-axis sensors have quite good performance in many applications including the wrist sensors in robot arms [13–15, 19], balances for wind tunnels [21–23] and precision assembly, etc. [24–25]

However, when applying a multi-axis force sensor into the vehicle wheel and make it to be a WFT, one critical issue is that the inertia effect of WFT can be non-ignorable due to the large mass [8]. Moreover, the WFT is typically mounted on the wheel of a moving vehicle, especially on a high speed car when abruptly accelerating, the mass/inertia of the transducer/wheel itself will have extra effect on the sensor response. The inertia loads will be detected and coupled into in the signal outputs as well, causing the sensor accuracy to decrease. Recently, this effect of inertia coupling is highly drawing more and more attentions of researchers [8, 26–27]. Herrmann et al. [28] have made an investigation on mechanical properties of WFT during different driving manoeuvres, inertial impact on data ouput was firstly identified. Afterwards, a joint project [29] on the SWIFT WFT of Kistler [30] was implemented that three different wheels conditions without WFT, with two WFTs and four WFTs were tested, respectively. It indicated the added mass/inertia of WFT had more effect on the spindle loads and accelerations compared to the effect on wheel-body displacements. Lately, another similar research was conducted by You [31] at MTS Corp [32]. The mass effect of WFT on vehicle dynamic response was analysed by using virtual modeling approach, so the frequency shifts and damage changes were pointed out. Until now, researches have revealed the mass/inertia indeed have impact on data output, that is, the inertia load under acceleration fields could reduce the output accuracy, however, no elegant solution is provided to restrain the effect yet, or the proposed signal processing for inertia decoupling still relys heavily on empirical models of road tests. One of the principal causes is that inertial coupling mechanism, including how and what extent it affects the strain measurement, is not intensively developed structurally and experimentally.

For the reasons presented above, our investigation will put the emphasis on the inertia coupling mechanism of a WFT from the aspect of structure decoupling in the first place. The inertia coupling can be performed using theoretical approach, and then it is verified by Finite Element Modeling (FEM) and experimental tests. In this paper, a practical case of six-axis WFT with an eight spoke structure is also introduced firstly so that the structure decoupling principle is determined. Based on the principle of equivalent mass and rotary inertia, inertia load analysis of the structure is performed, and therefore the inertia impact can be obtained. At last, inertia characteristics are identified and verified with comparative analysis of FEM results in simulation and calculated values in theory.

## Principle and Analysis

### Measurement principle of WFT

**Fig 1:**
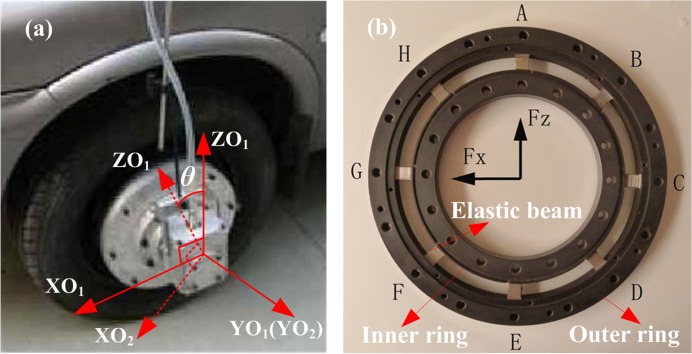
WFT developed at Southeast University (a) and the elastic body of the WFT (b).

Generally, the multi-axis WFTs have quite similar systems consisting of the mechanical structures, data acquisition and signal transmission system such as the typical WFTs developed at Southeast University [8–12] (see Fig. 1). Some design issues of WFT can be introduced for inertia coupling analysis.

**Coordinate transformation.** When applying a MFS to be a WFT in wheels, coordinate transformation exists between the spinning wheel and moving vehicle (Fig. 1). The vehicle Cartesian coordinate (O_1_-X_1_Y_1_Z_1_) can be transformed into wheel/WFT coordinate located in wheel center (O_2_-X_2_Y_2_Z_2_), the relation is given as

$$\left[\begin{array}{c} F_{x1}\\ F_{y1}\\ F_{z1}\\ M_{x1}\\ M_{y1}\\ M_{z1} \end{array}\right]=\left[\begin{array}{cccccc} \cos\theta & 0 & \sin\theta & 0 & 0 & 0\\ 0 & 1 & 0 & 0 & 0 & 0\\ -\sin\theta & 0 & \cos\theta & 0 & 0 & 0\\ 0 & 0 & 0 & \cos\theta & 0 & \sin\theta \\ 0 & 0 & 0 & 0 & 1 & 0\\ 0 & 0 & 0 & -\sin\theta & 0 & \cos\theta \end{array}\right]\left[\begin{array}{c} F_{x2}\\ F_{y2}\\ F_{z2}\\ M_{x2}\\ M_{y2}\\ M_{z2} \end{array}\right]$$ (1)

where *θ* is the rotating angle of the spinning wheel, subscript 1 and 2 represent the vehicle coordinates and the wheel coordinates, respectively.

As a result of real-time periodic signal with the transfer matrix, the generalized wheel force/moment on the wheel can be measured and computed. More importance is that if considering the wheel coordinate, all the deformations, stress and strain analysis can be carried out by the linear static method. Also the motion vectors as multi-axis accelerations of the WFT will be under the static state, and therefore the equivalent mass or rotary inertia can be used in load distribution analysis of the WFT through Newton’s law of inertia.

**Static structural analysis.** As the core part of a WFT, the elastic body structure (Fig. 1.b) consists of eight elastic beams, one inner ring and one outer ring. Strain gauges are attached to the elastic beams to sense deformation, and the two rings are used for force transmission between tyre and wheel hub, respectively. According to the theory of Material Mechanics, the hypothesis for stress-strain analysis can be listed essentially:

(1) Comparing to the small elastic beams, the two rings have large amount of mass and they will have the close fit joints attached to their connections (wheel hub or rim), so the two rings can be considered to be relatively rigid.
(2) In the process of elastic beams’ deformation, cross section perpendicular to the elastic beam keeps plane. Also no direct stress exists between the longitudinal fibers when shearing the beams.

The boundary condition of the eight elastic beams will be fixed-end with only supports shifting. Consequently, the inner ring is considered to be fixed and the outer ring has one free translation degree at motion direction, or vice versa. When the six-axis forces are applied to the WFT individually, stress and deformation analysis is as below:

As is the case for a single force at X direction *F*_*x*_, when it is applied to the WFT individually, beam C and G suffer the tensile and compressive deformations, respectively. Beam A and E are bending deformations, and the remaining beams B, D, F and H are combined deformations of tensile-compressive and bending, respectively. When a single force at Y direction *F*_*y*_ is applied to the WFT, all the beams will produce bending deformations. The case of *F*_*z*_ will be the same as *F*_*x*_ with 90 degrees rotation anticlockwise. Similarly, when a single moment *M*_*x*_ is applied, beams A and E are bending deformations, beams C and G are torsional deformations, and the remaining beams B, D, F and H are the combined deformation of bending and torsional deformations. When a single moment at Y direction *M*_*y*_ is applied, all beams produce bending deformations. The case of *M*_*z*_ is similar to *M*_*x*_ as well.

**Fig 2:**
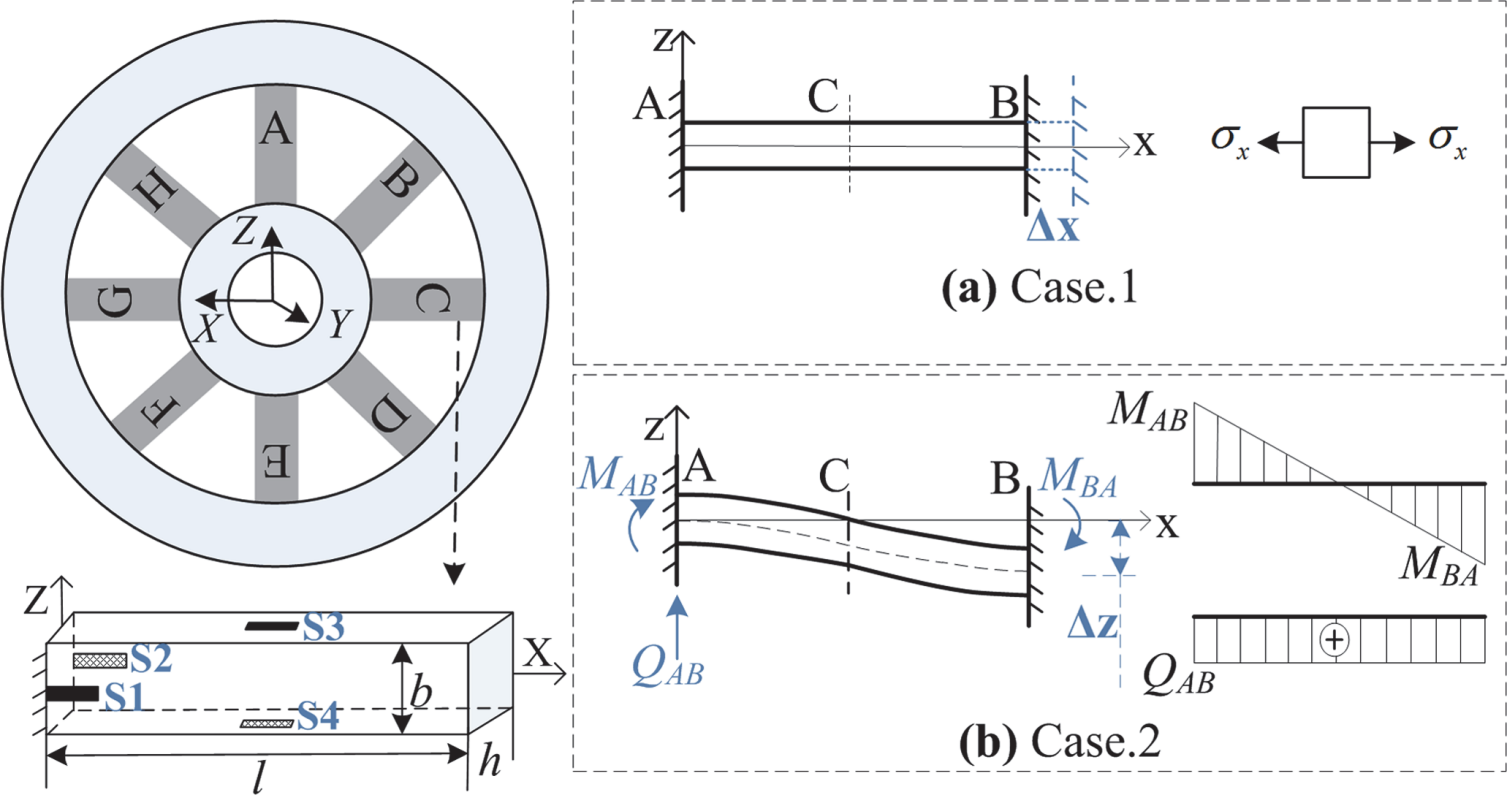
The elastic beam and the deformations. Case.1 represents the tensile and compressive deformation; Case.2 represents the bending deformation; S1~S4 represent the strain gauges.

According to the superposition principle in mechanics, the combined deformations can be decomposed into three basic deformation behaviors including tensile-compressive, bending, and torsional deformations. Since the measurement of torsional deformation always needs a couple of strain gauges with 45 rotations with respect to the beam axis, we can just select tensile-compressive and bending deformations (Fig. 2). This makes all the strain gauges be of longitudinal arrangement along each elastic beam so that the torsional effect is removed.

*Case.1*. Tensile-compressive deformation (Fig. 2.a). In this case, the elastic beam forms the uniaxial stressed state and the stress relation is given as

$$F_{x}=\frac{EA}{l}\Delta x=\sigma_{x}\cdot A$$ (2)

where *E* is elastic modulus, *σ*_*x*_ is the stress, *l* is the length of the beam and *A = bh* is the cross sectional area

*Case.2*. Bending deformations (Fig. 2.b). A relative displacement occurs between the inner and outer ring, rotation and axial displacement are restricted by the rigid rings. The rotation-displacement formula of the structure is given as

$$\left\{ \begin{array}{l} M_{AB}=-\frac{6EI}{l^{2}}\Delta z=M_{BA}\\ Q_{AB}=\frac{12EI}{l^{3}}\Delta z \end{array}\right.$$ (3)

where *M*_*AB*_ represents the bending moment, *Q*_*AB*_ represents the shearing force, and the *I = bh* ^*3*^ */12* is the geometrical moment of inertia.

Clearly, the stress is uniformly distributed along the beam in case.1. But in case.2, the antisymmetric bending load makes an inflection point on the deformation curve so that only shearing force will exist and the bending moment is approximately zero; the maximum bending moment occurs at the end of beam.

In consequence, for bending deformation in case.2, the strain-gauges can be arranged at the end of the beam, and the beam’s side where neutral axis exists can be unoccupied. For tensile-compressive deformation in case.1, discriminating measurement is taken into account so that strain gauges are arranged on neutral axis of the beam. Also a finite element verification of the pure theory analysis was provided by us [5]. The significance is that this detailed illustration gives the static strain and deformation analysis of the elastic body and it will be applicable for the inertia load analysis of the WFT as well.

**Self-decoupling approach.** The strain gauge based WFT here convert the signals with amplification. Wheatstone bridge circuits are used to convert the resistance changes into voltage outputs and further increase the sensitivity of the circuit. As a mathematical description, the vectors between applied load ***F*** (*F*_x_, *F*_*y*_, *F*_*z*_, *M*_*x*_, *M*_*y*_, *M*_*z*_) and the vector of the strains measured for each channel ***E***_S_ = (*E*_*Fx*_, *E*_*Fy*_, *E*_*Fz*_, *E*_*Mx*_, *E*_*My*_, *E*_*Mz*_) can be expressed as

$${\vec{F}}_{6\times 1}=C_{6\times 6}\cdot {\vec{E}}_{6\times 1}$$ (4)

Generally, if matrix [C] is diagonal with all zero off-diagonal values, the sensor will be completely self-decoupled so that the output of a particular strain signal corresponding to a specific force component is not affected by the application of any other force components. In this case, [C] is a constant square matrix referred to as the sensor stiffness matrix, strain sensitivity and the amplification factors, and Eq. (1) can be simplified as

$$F_{i}=C_{ii}\cdot E_{i}\;,i=1\ldots 6(C_{ij}=0,i\neq j)$$ (5)

**Fig 3:**
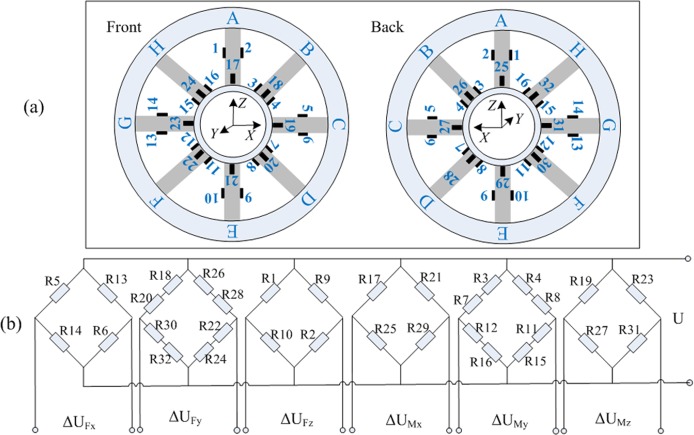
Strain gauge arrangement (a) and the Wheatstone bridge connection mode (b). The numbers of arrangement accord with that of Wheatstone bridge, representing strain gauges.

According to the strain and deformation analysis, full-bridge circuits are built where strain gauges connect to one bridge per load component (Fig. 3). The strain gauges at the end of each beam sense the bending deformations, and gauges on neutral axis of the beam sense the tensile-compressive deformations. Totals of 32 same strain gauges are hard-wired into six Wheatstone bridge. The strain output of each channel can be evaluated as the averages of the strains in the measurement bonding

$$\{\begin{array}{c} \begin{array}{c} \begin{array}{c} E_{1j}=0.25\;{\left(\varepsilon_{5}+\varepsilon_{6}-\varepsilon_{13}-\varepsilon_{14}\right)}_{j},\;j=1\ldots 6\\ E_{2j}=0.125{\left(\varepsilon_{18}+\varepsilon_{20}+\varepsilon_{22}+\varepsilon_{24}-\varepsilon_{26}-\varepsilon_{28}-\varepsilon_{30}-\varepsilon_{32}\right)}_{j},\;j=1\ldots 6 \end{array}\\ E_{3j}=0.25\;{\left(\varepsilon_{1}+\varepsilon_{2}-\varepsilon_{9}-\varepsilon_{10}\right)}_{j},\;j=1\ldots 6 \end{array}\\ E_{4j}=0.25\;{\left(\varepsilon_{17}+\varepsilon_{29}-\varepsilon_{21}-\varepsilon_{25}\right)}_{j},\;j=1\ldots 6\\ E_{5j}=0.125{\left(\varepsilon_{3}+\varepsilon_{7}+\varepsilon_{11}+\varepsilon_{15}-\varepsilon_{4}-\varepsilon_{8}-\varepsilon_{12}-\varepsilon_{16}\right)}_{j},\;j=1\ldots 6\\ E_{6j}=0.25{\left(\varepsilon_{19}+\varepsilon_{31}-\varepsilon_{23}-\varepsilon_{27}\right)}_{j},\;j=1\ldots 6 \end{array}$$ (6)

where strain *j* denoted the corresponding application of each individual force component.

This determined connection modes of the bridge not only completely eliminate the coupling errors between the six-axis in theory, which means each applied force affects only the output of the corresponding bridge while the outputs of the other bridges are irrelevant [9], the temperature compensation is also be achieved to a certain extent by the full-bridge circuits. To obtain the self-doubling relationship of  Eq.(6), the strain measurement for each individual load is analyzed as bellow. As for the symmetrical structure of the sensor, the analysis of applied load at X direction (*F*_*x*_ and *M*_*x*_) are same to the one at Z direction (*F*_*z*_ and *M*_*z*_) with 90 degrees rotation anticlockwise.

(1). Applying the Force F_x_ along X axis. Suppose that an individual force *F*_*x*_ is applied to the sensor along X axis positively, beam C suffers tensile deformation and beam G suffers compressive deformation. *F*_*x*_ is measured by the output between the strain of R5 and R6 and the strain of R13 and R14, and so *E*_*Fx*_ = *E*_*11*_ = [(*ε*_*5*_+*ε*_*6*_)-(*ε*_*13*_+*ε*_*14*_)].

As for the cross coupling output of *F*_*y*_, beams B, D, F and H are all the combined deformations including bending and tensile-compressive deformations. Under the tensile-compressive deformations, the strains of gauges R18, R20, R26 and R28 and the strains of gauges R20, R24, R30 and R32 cancel each other out. Under bending deformations, the strains of gauges R18, R26, R24 and R32 and the strains of gauges R20, R28, R22 and R30 cancel each other out. Therefore, *C*_*Fy*_ = *C*_*21*_≈0. For the cross coupling *F*_*z*_, beams A and E suffer the bending deformations (Case 2); the strain gauges R1, R2, R9 and R10 are zero approximately, so the *E*_*Fz*_ = *E*_*31*_≈0. For the cross coupling *M*_*x*_, the strain gauge R17 and R25 and strain of gauge R21 and R29 will cancel each other out, as they locate at neutral axis of the beam A and E under the bending deformations. so *C*_*Mx*_ = *C*_*41*_≈0. For the cross coupling *M*_*y*_, the strains of gauges R3, R4, R7 and R8 and the strains of gauge R11, R12, R15 and R16 cancel each other out under the tensile-compressive deformation; the strains R3, R8, R12 and R15 and the strains R4, R7, R11 and R16 cancel each other out under the bending deformations. So, *C*_*My*_ = *C*_*51*_≈0. For the cross coupling *M*_*z*_, the strain of R19 and R31 under tensile deformation of beam C will cancel out the strain of R23 and R27 under compressive deformation of beam G. so *C*_*Mx*_ = *C*_*41*_≈0.

(2). Applying the Force F_y_ along Y axis. In this case, all the eight beams suffer bending deformations. Only the strain gauges at the surface of each elastic beam (R17~R32) produce non-zero values while the values of the rest gauges (R1~R16) at neutral layer are approximately zero. (Fig.3 and the Case.2). As a result of Eq.(6), *F*_*y*_ is measured by the strains output of R18, R20, R22 and R24 and the strains of R26, R28, R30 and R32 with the eight times of a single strain value. So, *E*_*Fy*_ = *E*_*22*_ = (*ε*_*18*_+*ε*_*20*_+*ε*_*22*_+*ε*_*24*_-*ε*_*26*_-*ε*_*28*_-ε_*30*_-*ε*_*32*_).

The strain gauge R17 and R21 and strain of gauge R25 and R29 will cancel each other out under the bending deformations of beam A and E. So, *C*_*Mx*_ = *C*_*42*_≈0. Likewise, the strain gauge R19 and R23 and strain of gauge R27 and R31 cancel each other out with *C*_*Mz*_ = *C*_*62*_≈0. However, the rest cross coupling *F*_*x*_, *F*_*z*_ and *M*_*y*_ will be all to be eliminated with *E*_*12*_ = *E*_*32*_ = *E*_*52*_≈0 due to the gauges bonding to the neutral axis.

(3). Applying the Force M_x_ around X axis. Suppose that an individual *M*_*x*_ is applied around X axis in a counterclockwise direction, beams A and E suffer bending deformations. *M*_*x*_ is measured by the compressed strain gauges R17 and R29 along the beams and the stretched R21 and R25. So *E*_*Mx*_ = *E*_*44*_ = [(*ε*_*17*_+*ε*_*29*_)-(*ε*_*21*_+*ε*_*25*_)].

For the cross coupling *F*_*x*_, beam C and G only suffer the same torsional deflection and all the strains of R5, R6, R13 and R14 produce the same result, then *E*_*Fx*_ = *E*_*144*_ = (*ε*_*5*_+*ε*_*6*_-*ε*_*13*_-*ε*_*14*_) ≈0. For the cross coupling *F*_*y*_, beams B, D, F and H are all affected by the combined deformation of torsional and bending deformation. The torsional effect in strain gauge of these beams are all same to be eliminated. The bending components in the gauge R18, R24, R28 and R30 are compressed but R20, R22, R26 and R32 are stretched, and they cancel out each other by *E*_*Fy*_ = *E*_*24*_ = (*ε*_*18*_+*ε*_*20*_+*ε*_*22*_+*ε*_*24*_-*ε*_*26*_-*ε*_*28*_-ε_*30*_-*ε*_*32*_)≈0. For the cross coupling *F*_*z*_, beams A and E suffer the bending deformation. The strains of R1, R2, R9 and R9 are approximately zero due to their location of neutral layer. Then, *E*_*Fz*_ = *E*_*34*_≈0. For the cross coupling *M*_*y*_, beams B, D, F and H are all affected by the combined deformation of torsional and bending deformation. As mentioned above, the torsional effect is eliminated. The bending components of R3, R4, R7, R8, R11, R12, R15 and R16 are approximately zero due to the neutral layer effect, and then *E*_*My*_ = *E*_*54*_≈0. For the cross coupling *M*_*z*_, beam C and G only suffer the same torsional deflection and the effect in strain gauge R19, R23, R27 and R31 are all same to be eliminated with *E*_*Mz*_ = *E*_*56*_ = (*ε*_*19*_+*ε*_*31*_-*ε*_*23*_-*ε*_*27*_) ≈0.

(4). Applying the Force M_y_ around Y axis. Suppose that an individual *M*_*y*_ is applied around Y axis in a counterclockwise direction, all the eight beams suffer bending deformations. The values of the strain gauges (N17~No.32) at neutral layer are approximately zero. The values of the strain gauges (R1, R2, R5, R6, R9, R10, R13 and R14) are also approximately zero due to they located at the middle of the beams under bending deformation (Fig. 3 and the Case.2). As a result of Eq.(6), the cross coupling outputs in *F*_*x*_, *F*_*y*_, *F*_*z*_, *M*_*x*_ and *M*_*z*_ are all approximately zero with *E*_*15*_ = *E*_*25*_ = *E*_*35*_ = *E*_*45*_ = *E*_*65*_≈0.

However, the rest eight strain gauges may produce non-zero values. *M*_*y*_ is measured by the strains output of the stretched R3, R7, R11 and R15 and the strains of the compressed R26, R28, R30 and R32 with the eight times of a single strain value. So, *E*_*My*_ = *E*_*55*_ = (*ε*_*3*_+*ε*_*7*_+*ε*_*11*_+*ε*_*15*_-*ε*_*4*_-*ε*_*8*_-ε_*12*_-*ε*_*16*_).

### Analysis of inertial impact

**Inertia load distribution.** To resolve the inertia influence on WFT, multi-axis accelerations are investigated individually based on superposition principle. According to Newton’s law of inertia, the generalized inertia load is equivalent to the acceleration (or angular acceleration) multiplied by mass (or rotary inertia), so the inertia load distribution can be derived by static method. Since the inner ring of the elastic beam is considered to be a fixed-end, the outside parts such as the outer ring, rim, tyre and other mountings will be the effective inertia/mass.

It should be noticed that the proposed configuration of an eight-spoke elastic body in WFT can be considered to be a doubled Maltese crossbar, which means the applied approach using Newton’s law with equivalent principle can also generally fit some other MFS as long as a similarly regular configuration exist.

(1). Inertia load at Y direction. As is the case for a single acceleration at Y direction *a*_*y*_, when it is applied to the WFT, an inertia force *ΔF*_*y*_ can be uniformly distributed on eight elastic beams with bending deformations. Each beam will have an extra output coupled into *F*_*y*_ signal with

$$\Delta F_{y}\text{=}ma_{y}/8=f_{i}$$ (7)

where *m* is the effective inertia-mass outside the elastic beams, *i = A*,.,*H* represent beam number.

Similarly, when the angular acceleration at Y direction *ẇ*_*y*_ is applied individually, an inertia moment *ΔM*_*y*_ is uniformly distributed with bending deformations. Replacing *m* with rotary inertia *J*_*y*_ about Y axis, Eq. (7) can be the inertia load of angular acceleration at Y direction for each beam.

(2). Inertia load at X or Z direction. When the acceleration *a*_*x*_ is applied individually, the load distribution is

$$\Delta F_{x}=m\cdot a_{x}=2f_{A}+2f_{C}+4f_{B}$$ (8)

where subscript A, B and C represent the beam number, respectively. The inertia load is distributed with tensile-compressive deformations on beam C and G, bending deformations on A and E, combined deformations of tensile-compressive and bending on the remaining B, D, F and H beams, respectively. Therefore, the displacements can be given by Hooke's law as

$$\left\{ \begin{array}{l} \Delta l_{A}=\frac{f_{A}l^{3}}{12EI}\\ \Delta l_{B}=\frac{f_{B}l}{2EA}+\frac{f_{B}l^{3}}{24EI}\\ \Delta l_{C}=\frac{f_{C}l}{EA} \end{array}\right.$$ (9)

As the two rings are considered to be rigid body, beams A, B and C will have equivalent displacements at X direction with the compatibility equation as

$$\Delta l_{A}=\Delta l_{B}=\Delta l_{C}$$ (10)

Substituting the Eq. (8) and (9) into the Eq. (10), the inertia load distribution is obtained as

$$f_{C}=\frac{Al^{2}}{12I}f_{A}=f_{B}\left(\frac{1}{2}+\frac{Al^{2}}{24I}\right)\text{=}\lambda ma_{x}$$ (11)

where *λ* is the coefficient with respect to *b*, *h* and *l*.

The case of *F*_*z*_ will be same to *F*_*x*_ with 90 degrees rotation anticlockwise. Likewise, when the angular acceleration is applied individually, the inertia load can be given by replacing force *ΔF* with moment *ΔM*. Using the same analytical method according to the above steps, inertia moment distribution of *ΔM*_*z*_ and *ΔM*_*x*_ for each beam can be solved as well.

Now, the inertial load distribution of the elastic body under the multi-acceleration fields can be identified and calculated using the Eq. (7) ~ Eq. (11). For each elastic beam, these inertia loads will produce extra strains in the outputs. According to Eq. (2) in case.1 and Eq. (3) in case.2, stress-strain and deformations of the inertial loads can be also easily calculated by the equation of *σ = Eε*.

**Inertia decoupling output.** Since the WFT is self-decoupled due to the strain gauge arrangement, inertia interference of elastic beams will be also reduced by the Wheatstone bridge to some extent. However, the remaining inertia errors may be transmitted and amplified by the calibration coefficient *G* because the calibration is always performed under static condition. The model of multi-axis WFT with inertia decoupling can be obtained by rewriting Eq. (4) as

$$F_{r}=F_{o}-\Delta f_{ine}=G_{i}(\sum S-\sum S_{ine})=K_{i}\sum S$$ (12)

where *F*_*r*_ is the true value of the wheel generalized force, *F*_*o*_ is the six output of WFT, *Δf*_*ine*_ is the inertia component which is coupled into the output, *K*_*i*_ is the modified calibration coefficient, *ΣS*_*ine*_ is the effective strain output of inertia component in theory which needs to be determined in the following.

(1). Inertia coupled interference at Y direction. The inertia load *ΔF*_*y*_ will have effect on all 32 strain gauges with bending deformations of each elastic beam, but only strain gauges (No.17~24 and No.24~32) on the dual surface of the beams have inertia components coupled into *F*_*y*_ output. The remaining gauges could be easily compensated by bridge circuits (See Fig. 3.b). Therefore the extra strains which caused by inertia load in the output will have a total value of 8*Δε*_*Fy*_ with

$$\Delta \varepsilon_{Fy}=\frac{\Delta \sigma_{Fy}}{E}=\frac{0.5\Delta F_{y}l}{EW_{t}}=\frac{3ma_{y}l}{8Ebh^{2}}$$ (13)

where *W*_*t*_ *= 2I/h* is section modulus in bending, respectively.

Similarly, the inertia moment *ΔM*_*y*_ will make the strains coupled on strain gauges No.3, 4, 7, 8, 11, 12, 15 and 16 with a total value of 8*Δε*_*My*_ where

$$\Delta \varepsilon_{My}=\frac{\Delta \sigma_{My}}{E}=\frac{3J_{y}{\dot{\omega }}_{y}}{4Ehb^{2}}$$ (14)

(2). Inertia coupled interference at X or Z direction. In the case of inertia load *ΔF*_*x*_, different deformations occur on beam C and G, beam A and E, and beam B, F, D and H, respectively. The deformations of beam A and E will have no influence on their strain gauges (No.1, 2, 9, 10, 17, 21, 25 and 29) due to longitudinal or neutral axis arrangement. Though the combined deformation of tensile-compressive and bending occurs on beam B, F, D and H with strain gauges (No.18, 20, 22, 24, 26, 28, 30, 32 and No.3, 4, 7, 8, 11, 12, 15, 16), the coupled strains caused by inertia can be compensated by symmetrical structure and bridge circuits yet. Only the beam C and G with tensile-compressive deformations may have interference output on strain gauges of No.5, 6, 19, 27, 13, 14, 23 and 31. According to Fig. 3.b, the extra strain which caused by inertia load *ΔF*_*x*_ is total of 4*Δε*_*Fx*_ where

$$\Delta \varepsilon_{Fx}=\frac{\Delta \sigma_{Fx}}{E}=\frac{f_{C}}{EA}\text{=}\frac{\lambda ma_{x}}{Ebh}$$ (15)

Similarly, applying the same analysis procedure to the angular acceleration case, the extra strains which caused by inertia moment *ΔM*_*x*_ will have effects on strain gauges No.17, 25, 21, and 29. The result total value is 4*Δε*_*Mx*_ in which

$$\Delta \varepsilon_{Mx}=\frac{\Delta \sigma_{Mx}}{E}=\frac{kJ_{x}{\dot{\omega }}_{x}}{Ebh^{2}}$$ (16)

The coefficient *k* is related to *b*, *h*, *l* and *μ* (the poisson ratio). Apparently, the inertia component for *ΔF*_*z*_ and *ΔM*_*z*_ will equal to Eq. (15) and (16) by replacing x with z, respectively.

If the motion information of three-axis accelerations and three-axis angular accelerations can be detected by such sensor unit as a six-axis MEMS sensor, the strain and deformation of the WFT under inertia loads will be easily calculated by Eq. (13) ~ Eq. (16). Substituting the strain values of inertia errors into the Eq. (10) and Eq. (4), the multi-axis wheel forces/moments with inertia decoupling will be recorded in real time for the vehicle.

## Results and Verification

### Numerical simulation using FEM

**Fig 4:**
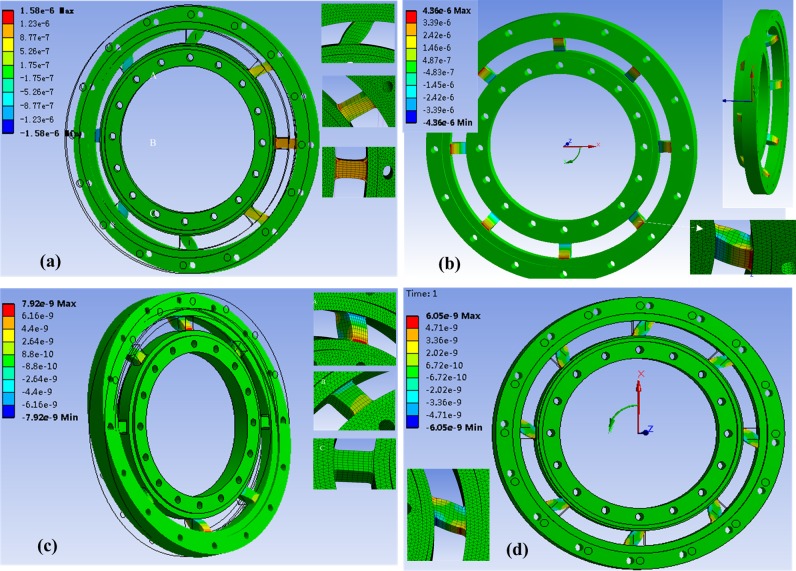
FEM results of inertia load individually. The symbol (a), (b), (c) and (d) represent inertial load Δ*F*_*x*_, Δ*F*_*y*_, Δ*M*_*x*_ and Δ*M*_*y*_ respectively. Each inset figure shows the detailed information of deformation.

According to the theoretical analysis, inertial errors cannot be completely compensated by the strain gauge arrangements and Wheatstone bridges because the inertia loads make different deformation and strain output on the elastic body. Fortunately, the elastic body structure of the WFT can be resolved, formula deduction gives the inertia coupling mechanism under multi-axis accelerations. To test and verify the manual derivation, a set of parameters for the WFT can be given as: an 175/65-R14 tyre with the effective rolling radius of 284mm, the total wheel mass of about 36.0kg including 6.0kg modified rim, 5.2kg wheel hub, 10.03kg tire and 14.52kg WFT, etc. The measured wheel rotational inertia I_y_ = 1.88kgm^2^, I_x_ = I_z_ = 0.92kgm^2^ where x, y and z represent the rotation axis. The average maximum accelerations are about 4m/s^2^ and 14.1rad/s^2^ which corresponding to the first gear for a passenger car. Calculated loads are approximate 150N for the three inertia forces, 30N⋅m for inertia moment at Y direction, and 15N⋅m for inertia moment at X and Z direction, respectively. Applying the loads to the elastic body of WFT, finite element method (FEM) calculation can be performed (shown in Fig.4).

Results indicate that the deformations and strain of elastic beam agree well with the analytical inertia load distribution. For inertia load *ΔF*_*x*_, the normal strain of beam C, B and A decrease in sequence at X direction. The maximum strain value occurs on beam C and G with tensile-compressive deformations while the strains on beam A and G with bending deformations are much small. For inertia load *ΔF*_*y*_, all the beams have same bending deformations with the maximum value at the end of the beam. For inertia load *ΔM*_*y*_, all the beams have same bending deformations which are similar to the case of inertia load *ΔF*_*y*_. Likewise, for inertia load *ΔM*_*x*_, the normal strain of beam A, B and C decrease in sequence at X direction. A maximum strain value on beam A and E is observed while the beam C and G have much small strains. Detailed information of beam can refer to the inset figures in Fig. 4 as well.

By substituting the parameters into theoretical formulas and extracting the strains in FEM simulation, quantitative and comparative analysis of inertia coupling errors can be achieved. Table 1 shows the strain value under each inertia load individually (values at X and Z direction are equal). Also the relative error between theoretical calculation and the FEM simulation can be defined as

$$r=\frac{\left|v_{t}-v_{s}\right|}{v_{s}}\times 100\%$$ (17)

where *v*_*t*_ represents the values of theoretical derivation and *v*_*s*_ represents the values of FEM simulation.

**Table 1:** The strain values under inertia loads.

**Inertia load**	**ΔF**_**x**_ (**ΔF**_**z**_)	**ΔF**_**y**_	**ΔM**_**x**_ (**ΔM**_**z**_)	**ΔM**_**y**_	
**Theoretical calculation**	1.21e-6	4.12e-6	6.95e-9	5.67e-9
**Simulation result**	1.18e-6	4.25e-6	7.22e-9	5.85e-9
**Errors (%)**	2.5	3.1	3.7	3.1

Results indicate that the strain values in FEM simulation agree well with the values of the theoretical derivation for each inertia load. Since all the relative errors are less than 5% which are within acceptable, it confirmed that the theoretical derivation is correct. However, the errors are understandable and foreseeable not only because the hypothesis in the pure theoretical derivation exists, but also because of chamfering geometric of structure, mesh quality of generation or other constraint reasons in FEM simulation. Nevertheless, if considering a practical measurement range of 10000N and 4000N⋅m, the inertia loads will have the maximum impact on the signal output about 1.5%.

### Experimental verification

The derived formulation considers the assumption that all the inertial loads are transmitted into a static or quasi-static based on the principle of equivalent mass and rotary inertia. As the calculated results cannot meet real conditions with many approximate or equivalent values, the inertia coupling errors need to be verified and tested under six-axis acceleration fields. The tested mass/inertial parameter will be also used for inertial decoupling according to Eq.(16). Due to the practical difficulties and economic reasons, the large six-axis acceleration simulation equipment is improbably built for us, but in fact, a three-axis self-decoupled WFT will be adequate for the normal commercial vehicles [9]. Therefore, the three-axis inertial load including *ΔF*_*x*_, *ΔF*_*z*_ and *ΔM*_*y*_ will be verified herein. A power motor will be used to drive the installed WFT to produce the angular acceleration field and a centrifuge can generate a steady state linear acceleration field in virtue of its centripetal force.

**Fig 5:**
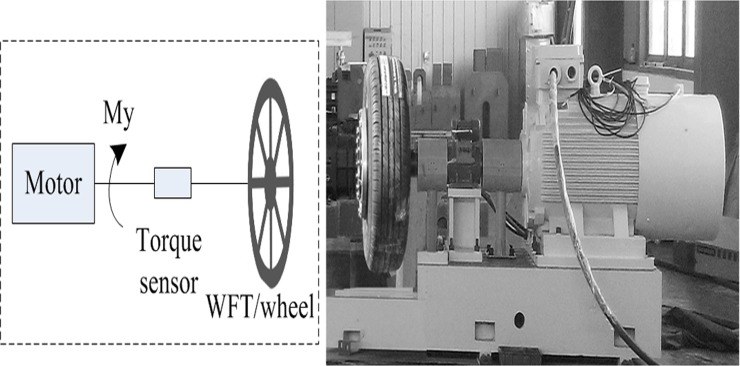
Wheel inertia device used for *ΔM*_y_ testing under angular acceleration.

**Fig 6:**
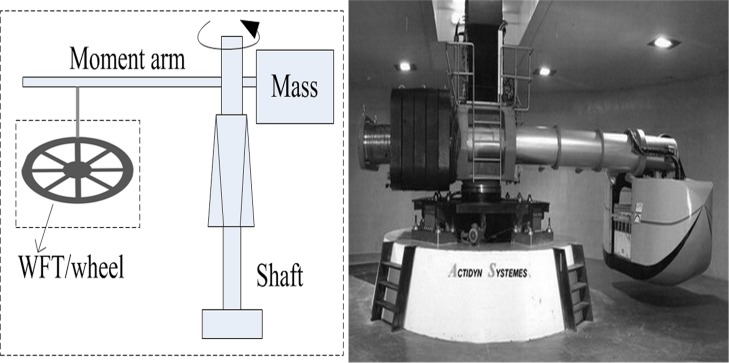
Geotechnical centrifuge used for *ΔF*_x_ and *ΔF*_z_ tests under linear acceleration.

Fig.5 shows a special tailored test device where a power motor, a torque sensor, a transmission shaft and the WFT assembly are included. The whole WFT is installed on wheel to simulate real conditions. By controlling the accelerating time from zero to the rated angular velocity with a linear function generator, different angular accelerations is obtained. If being so, inertial moment *ΔM*_*y*_ can be applied to the WFT and can be fed back by a torque sensor of the motor. Fig. 6 shows the principle of how to generate a linear acceleration field by using the geotechnical centrifuge [33]. For security reasons, the centrifuge belonging to an engineering research institute of China cannot be made public but a similar description is shown. The assembled WFT is installed on the centrifugal arm via bolts. When the centrifuge is rotating under a constant angular speed, a centripetal force will be generated along the moment arm. By testing the WFT with different angular speed or the length of the arm, the inertial impact on the WFT will output.

**Fig 7:**
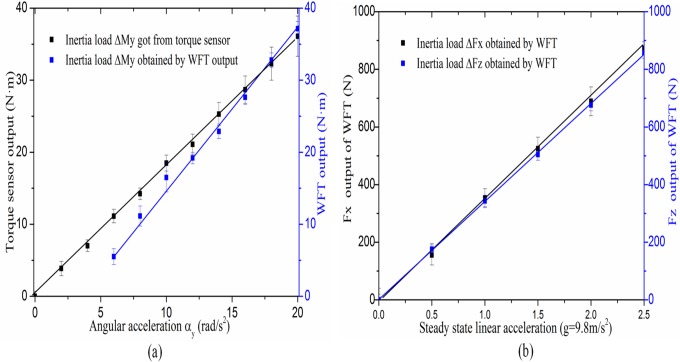
Experimental results of inertial load. The signal outputs of inertial moment *ΔM*_*y*_ (a) and that of inertial forces *ΔF*_*x*_ and *ΔF*_*z*_ (b) are expressed as functions of the accelerations, respectively. Curves are given as polynomial fitting based on original point data.

Fig. 7 shows that the wheel force output and moment output are expressed as functions of the accelerations, respectively. It indicates that when the acceleration is increasing, the inertia load indeed have signal coupling output which can be detected by the sensor or WFT. Also the relationship between the applied acceleration and inertia load for both wheel force and moment is the approximate linear, respectively. Giving an insight into the linear fitting curves, the slopes represent the inertial-mass or rotational inertia of the wheel to some extent. The calculated values are 2.11 for inertia moment *ΔM*_*y*_, 351.2 for inertia force *ΔF*_*x*_ and 340.1 for inertia force *ΔF*_*z*_. The results are corresponding to the measured rotational inertia of 1.88kgm^2^ and wheel mass of 36.0kg. The deviation errors may be caused by the mass such as the inner ring, the elastic beams of the elastic body, spindle of the test device or other parts which are not included in WFT output. Also, the deviation of inertia moment *ΔM*_*y*_ between torque sensor output and WFT output is a little larger than the deviation of inertia force between *ΔF*_*x*_ WFT output and *ΔF*_*z*_ WFT out. Because of different locations of the equipment in *ΔM*_*y*_ testing, the inertial mass for the torque sensor is a little larger than the inertial mass for WFT.

### Results of road test

**Fig 8:**
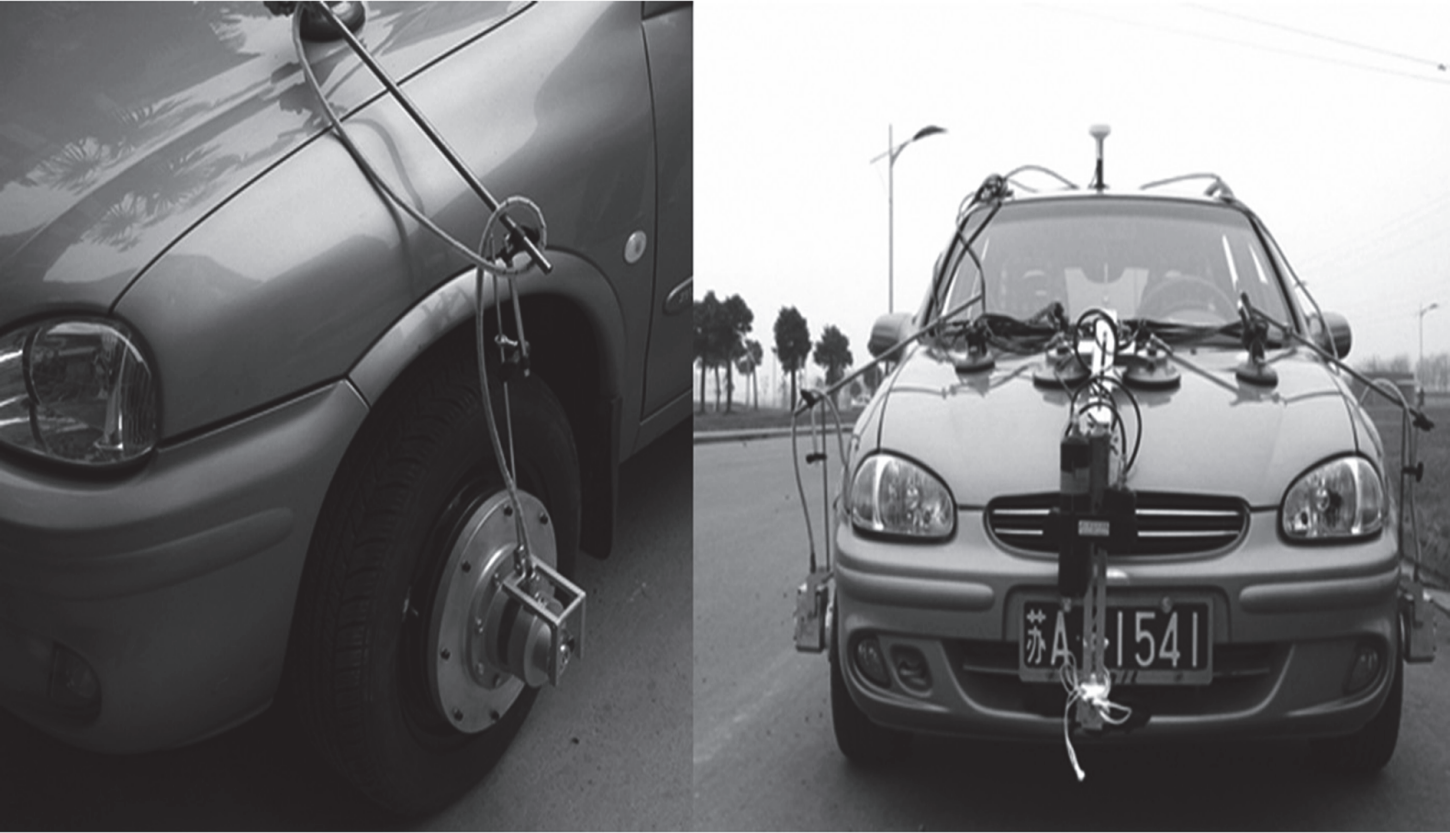
The vehicle with the WFT in road test.

**Fig 9:**
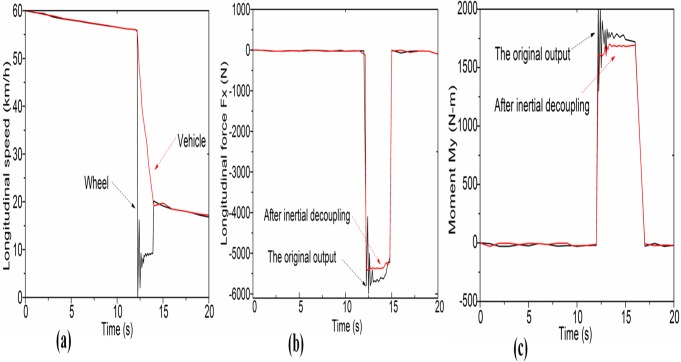
The result of a braking process test. The longitudinal speed (a), the longitudinal force *F*_*x*_ (b) and the drive moment *M*_*y*_ (c) as functions of the time, respectively.

To evaluate the inertial decoupling result of the proposed three-axis WFT in practical applications, the designed WFT is mounted on the left front wheel of a Chevrolet Sail vehicle with a manual transmission, as shown in Fig. 8. Meanwhile, some auxiliary instruments including a photoelectric five-wheel meter, a wheel speed sensor and an accelerometer are adopted to measure the longitudinal speed of the vehicle and wheel. To interpret the application of the inertial decoupled WFT, a braking process is performed when recording the tractive force and driving moment applied to the wheel. Fig. 9 shows the result of a routine asphalt road test. The car is firstly driving to a constant speed about 60km/h, and then beginning to coast with the neutral mode shifted. After that, the braking is applied at the 13^th^ second and release at 15^th^ second in favor of the anti-lock braking control system.

As shown in Fig. 9, we see that the longitudinal speed of the vehicle and the wheel both declined dramatically in this time period, and importantly, the wheel speed suddenly dropped but not to zero because of the activated anti-lock system. Therefore, the wheel suffers a resultant force of both sliding (denotes as *F*_*slide*_) and rolling resistance (denotes as *F*_*roll*_). This resultant force of the tyre is the longitudinal force of the vehicle, coming from the inertial impact of the whole vehicle in such a braking process. However, the WFT will output a result which expressed as

$$\left\{ \begin{array}{l} F_{x}=F_{car}+F_{in-WFT}=(F_{slide}+F_{roll})+F_{in-WFT}\\ M_{y}=M_{car}+M_{inertia}=M_{car}+M_{in-WFT} \end{array}\right.$$ (18)

where the subscript *car* represents the real data which will be of value for researchers but the component with subscript *in-WFT* is the error resulting from the inertial impact of WFT itself. The inertial decoupling of WFT is simply to subtract an average value of this error component by using Eq.(16). Fig. 9.b compares the longitudinal force *F*_*x*_ in the process with and without the inertial decoupling, and (c) compares the drive moment M_y_, respectively. Results show that the decoupled data is a little smaller than the original data after eliminate the added mass/inertial effect of WFT. After removing the inertial effect, the curves of WFT output also become smoother because the beginning jitter error of the anti-lock braking is also eliminated to some extent. Therefore, the WFT precision can be improved by reducing this inertial effect.

## Conclusion

In conclusion, we have confirmed the inertia coupling characteristics induced from multi-axis accelerations for the WFT. The inertial loads can be performed by the proposed theoretical approach which is verified by FEM analysis. Experimental verification is performed on the inertial loads *ΔF*_*x*_, *ΔF*_*y*_ and *ΔM*_*y*_ for the WFT as well. Several useful conclusions can be obtained as bellow:

(1) Though the proposed WFT is self-decoupled, the inertial loads cannot be compensated completely due to the structure and strain gauge arrangements.
(2) Inertia coupling mechanism of a WFT can be analyzed by the equivalent principle of inertia load, so that the inertia load distribution can be derived by static method.
(3) From the aspect of structure analysis, the inertial errors can be calculated with manual derivation. With the theoretical calculation, the equations may be coded into decoupling program in future signal processing and software algorithms for the real time output.
(4) A road test illustrates that the inertial decoupling is necessary and effective for a WFT.

As the inertia loads have inevitable impact on the signal output, it will be of great significance for accuracy improvement of WFT. The generalized method and the derived formula in this paper not only give an insight into the inertia coupling mechanism of WFT, but also is used for correction of the calibration coefficient which obtained under static condition.
